# A Case of Clozapine-Induced Hepatotoxicity: Management Considerations and Future Direction

**DOI:** 10.7759/cureus.70788

**Published:** 2024-10-03

**Authors:** Matthew B Bulkley, Kendahl Oberdorfer, Renee R Maan

**Affiliations:** 1 Psychiatry, Wayne State University School of Medicine, Detroit, USA; 2 Psychiatry, Henry Ford Health System, Detroit, USA

**Keywords:** clozapine, clozapine side effect, drug-induced liver injury (dili), hepatotoxicity, treatment-resistant psychosis

## Abstract

Clozapine is an atypical antipsychotic used for treatment-resistant schizophrenia. Its use is often limited due to its well-known association with a variety of side effects. Hepatotoxicity is a less common side effect and has been infrequently reported. Here, we present the case of a patient who developed abdominal discomfort and right upper quadrant pain after clozapine was initiated. Liver transaminases were found to be elevated and continued to rise despite cessation of another psychiatric medication more commonly associated with hepatotoxicity. Discontinuation of clozapine resulted in relief of symptoms and normalization of liver enzymes without any complications.

## Introduction

Clozapine is a second-generation antipsychotic used for the treatment of psychotic disorders, particularly treatment-resistant schizophrenia. Despite its effectiveness, clozapine is often used as a second-line agent due to its association with a variety of rare but serious side effects [1]. Agranulocytosis is one of the most well-documented side effects of clozapine, and frequent blood draws allow for careful monitoring of the white blood cell count [2]. However, clozapine use is also associated with other serious side effects that are less common and thus not monitored as closely. These include myocarditis, gastrointestinal obstruction, and hepatoxicity [2].

Hepatotoxicity is a rare side effect of clozapine that is not frequently reported [3]. Indeed, the incidence of liver enzyme abnormalities in clinical trials for clozapine was reported to be 1% [4]. Interestingly, in clinical practice, liver transaminases commonly rise to two to three times the upper limit of normal when initiating clozapine. This phenomenon is usually transient and asymptomatic and thus does not necessitate cessation of the drug [5]. However, in a small number of cases, drug-induced liver injury (DILI) may occur with transaminase levels rising above two to three times the upper limit of normal, requiring drug cessation and further workup. These cases often present with symptoms including abdominal pain, nausea and vomiting, jaundice, encephalopathy, and death in severe cases [6,7].

Here, we present the case of a patient with a treatment-resistant psychotic disorder initiated on clozapine. The patient was symptomatic with an elevation of liver transaminases greater than four times the upper limit of normal. Medical workup was consistent with drug-induced liver injury. Clozapine was promptly discontinued, resulting in the improvement of symptoms and normalization of liver transaminase levels.

## Case presentation

The patient is a 40-year-old African American male with a history of schizoaffective disorder, bipolar type, experiencing homelessness, who presented to the emergency department after being petitioned by police for auditory hallucinations. The patient’s medical history was not fully known, as records from other hospitals were not available. Upon admission, the patient was noted to be internally preoccupied with disorganized and bizarre behavior and delusions. The patient was medication non-adherent with home medications, which included valproic acid 1500 mg nightly, haloperidol 10 mg twice per day, and benztropine 1 mg twice per day. Pertinent lab results upon admission included normal liver enzymes (Table 1).

**Table 1:** Liver functional test trend during admission Summary of AST, ALT, and total and direct bilirubin levels during admission to a psychiatric facility. ^†^Valproic acid started. *Clozapine started. ^‡^Valproic acid discontinued. **Clozapine discontinued. ^§^Patient transferred to a medical facility. Reference ranges: AST, 11–39 U/L; ALT, 7–52 U/L; total bilirubin, 0.2–1.0 mg/dl; direct bilirubin, 0.0–0.3 mg/dl. AST: aspartate transaminase; ALT: alanine transaminase.

Admission day	Time	AST (U/L)	ALT (U/L)	Total bilirubin (mg/dl)	Direct bilirubin (mg/dl)
1^†^	6:30	32	13	0.3	-
22*	9:05	-	-	-	-
47	9:24	55	61	0.5	-
49^‡^	10:23	-	-	-	-
53	21:00	112	197	0.4	0.1
55**	11:44	129	246	0.4	-
56^§^	4:13	105	239	0.5	<0.1
56	21:53	71	183	0.5	0.1
58	8:59	46	147	0.5	0.1
63	6:30	22	45	0.7	0.1

The patient was initially started on paliperidone 6 mg, as that was the only medication he would consent to at the time. Paliperidone was titrated to 12 mg daily for psychotic symptoms. Shortly after, valproic acid 500 mg twice per day was started for mood dysregulation. Over the next week, the patient did not appear to be making any improvements. Thus, paliperidone was cross-tapered to fluphenazine, and valproic acid was titrated to 750 mg twice per day. Fluphenazine was increased to 12.5 mg twice per day, again without improvement in his symptoms. He remained verbally and physically aggressive, disorganized, paranoid, and sexually preoccupied. At that point, it was decided to cross-taper from fluphenazine to clozapine and add lithium, which was titrated to 600 mg twice per day.

The patient was initiated on clozapine 12.5 mg twice per day. Over the course of 25 days, clozapine was titrated to 175 mg twice per day. He began experiencing constipation 12 days after starting clozapine. The patient was given a bowel regimen to treat constipation, which included docusate, senna, and polyethylene glycol 3350 with little relief. He then began to complain of abdominal pain, mostly in the upper right quadrant, and nausea. The abdomen at the time was soft and non-tender to palpation. A comprehensive metabolic panel (CMP) was drawn three days later, and he was found to have elevated liver enzymes with an alanine transaminase (ALT) of 61 U/L and aspartate transaminase (AST) of 55 U/L (Table 1). At the time, valproic acid was considered to be the cause of the elevated liver enzymes due to its well-known association with liver toxicity and was discontinued.

The patient's symptoms persisted, and a CMP was rechecked six days later. Liver enzymes were found to have increased to an ALT of 197 U/L and an AST of 112 U/L (Table 1). Clozapine level was also checked and found to be 297 ng/mL. Due to concerns for decompensation in a difficult-to-treat patient with severe psychiatric symptoms and given that clozapine-induced hepatotoxicity is rare, our patient was continued on clozapine for one more day before rechecking liver functional tests (LFTs). At that point, LFTs had increased to an ALT of 246 U/L and an AST of 129 U/L (Table 1). It was determined that the elevated liver enzymes were possibly due to clozapine, as there were no other medication changes that could have explained the elevated liver enzymes.

The patient was then transferred to a medical hospital for further workup, and clozapine was abruptly discontinued. Lab findings during workup included a gamma-glutamyl transferase (GGT) of 424 U/L, normal iron studies, a white blood cell count (WBC) of 11,000 per microliter, a normal prothrombin time (PT), normal partial thromboplastin time (PTT), and normal international normalized ratio (INR), a negative hepatitis panel, a negative human immunodeficiency virus (HIV) antibody test, and a normal ceruloplasmin level.

**Figure 1:**
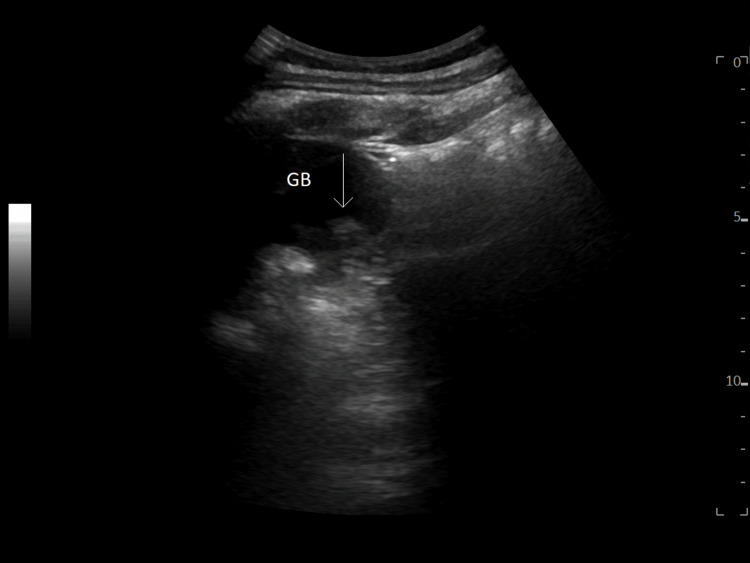
Ultrasound of the liver revealing hypoechoic structure in the gallbladder (later determined to be an artifact) without any other abnormalities. The arrow indicates the hypoechoic structure later determined to be an artifact. GB: gallbladder.

Following the discontinuation of clozapine, subsequent CMPs revealed liver enzymes began to decrease. A liver ultrasound demonstrated a hypoechoic structure within the gallbladder measuring 1.7 cm without other abnormalities. A follow-up ultrasound of the abdomen with Doppler did not demonstrate gallbladder abnormalities, and the previous structure identified was determined to be an artifact (Figure 1). The patient’s symptoms subsided within several days, and liver functional tests returned to normal seven days after discontinuation of clozapine (Table 1).

## Discussion

Here, we present a unique case of a symptomatic rise in liver transaminases in an individual with treatment-resistant schizophrenia after treatment with clozapine. Our initial approach was to discontinue valproic acid due to its well-known liver toxic effects. Indeed, 5-10% of patients on chronic valproic acid therapy develop liver enzyme derangements, which very well may progress to clinically apparent hepatotoxicity [8]. However, when transaminases continued to rise in our patient despite discontinuing valproic acid, we were concerned about another source of drug-induced liver injury and discontinued clozapine out of precaution. Further hepatic workup was conducted, which ruled out hepatitis, autoimmune hepatotoxicity, and other metabolic causes. Following discontinuation of clozapine, liver transaminases normalized within seven days. Given that the medical workup was negative for infectious, autoimmune, and metabolic causes of hepatic injury and since the discontinuation of clozapine was followed by normalization of liver enzymes, we determined clozapine to be the most likely cause of hepatotoxicity in this patient.

Clozapine is an especially effective antipsychotic for treatment-resistant psychosis and may be one of the only effective treatment options for certain patients [9]. The reasons for this are still not fully known, though they may be linked to clozapine’s unique mechanism of action. Like other atypical antipsychotics, clozapine exerts its effect by antagonizing dopamine D2 receptors and serotonin 5-HT2A receptors, but it is unique in that it has only a weak affinity for D2 receptors [10]. Given its weak affinity for D2 receptors, clozapine is less likely to induce extrapyramidal symptoms compared to other first- and second-generation antipsychotics [11]. However, clozapine does carry its own set of serious side effects, which is why it is reserved for treatment-resistant patients. Clozapine is associated with constipation, syncope, myocarditis, metabolic syndromes, seizures, and agranulocytosis [12,13]. In rare cases, clozapine has been reported to cause hepatotoxicity [3].

Patients initiated on clozapine commonly develop a transient raise in liver transaminases, specifically ALT. This rise in ALT usually does not exceed three times the upper limit of normal, typically resolves within a few weeks, and does not result in symptoms [5]. Our patient developed an ALT greater than three times the upper limit of normal with gastrointestinal symptoms, including abdominal pain, nausea, and RUQ pain, which is consistent with drug-induced liver injury [5]. Drug-induced liver injury is divided into intrinsic or idiosyncratic patterns. Intrinsic liver injury is typically dose-dependent and consistent among all individuals, such as in the case of acetaminophen toxicity [14]. Idiosyncratic liver injury typically only affects rare susceptible individuals and is more variable in presentation and timing of symptoms. Idiosyncratic liver injury is further divided into hepatocellular, cholestatic, and mixed patterns depending on lab results. Given the rarity and variability in the presentation of clozapine-induced hepatotoxicity, it seems to be more of an idiosyncratic mechanism rather than intrinsic [15]. In the case of our patient, elevation of AST and ALT but normal bilirubin levels reveal a hepatocellular mechanism of injury rather than a cholestatic or mixed pattern.

The mechanism of clozapine-induced hepatotoxicity is unknown, though its hepatic metabolism may contribute [5]. Clozapine is metabolized by the liver, primarily by the CYP1A2 enzyme. CYP1A2 converts clozapine into norclozapine, clozapine-N-oxide, and other hydroxylated metabolites [16]. Other enzymes known to metabolize clozapine include CYP2D6, CYP3A4, and CYP2C19 [17]. It is possible that the accumulation of one or more of clozapine’s metabolites produced during hepatic metabolism causes injury to hepatocytes, though further research is needed to elucidate the exact mechanism of clozapine’s rare hepatotoxic effects [5].

Prior to initiating clozapine, it is important to order a comprehensive metabolic panel to assess baseline liver function. Whether regular liver functional labs should be monitored during clozapine use remains debated, but given the low risk of hepatotoxicity, we believe it is only necessary to order liver functional labs if patients become symptomatic [14]. If patients begin experiencing gastrointestinal symptoms such as abdominal pain, nausea, or vomiting while on clozapine, as was the case with our patient, liver enzymes should be checked to evaluate for drug-induced hepatotoxicity. In the case of patients with elevated liver enzymes more than three times the normal limit, clozapine should be discontinued, and the patient should receive further workup as liver failure can develop [5]. Since clozapine-induced hepatotoxicity is so rare, other causes of hepatic injury should also be ruled out, including infectious hepatitis, autoimmune causes, metabolic diseases, and other iatrogenic sources. Thus, important labs to consider during workups include CBC, CMP, hepatitis panel, autoimmune panel, iron levels, and ceruloplasmin levels [13]. Any other medication the patient is taking should be investigated to determine if another drug could be contributing to liver toxicity or causing a drug-drug interaction. Imaging studies such as ultrasonography and computed tomography can also help rule out other etiologies of liver injury, including choledocholithiasis and malignancy [15].

Discontinuing clozapine after a patient experiences hepatotoxicity has not been studied, so no protocol is in place when discontinuation is needed [18]. Furthermore, since clozapine is often used after a patient has already failed multiple antipsychotics, it is difficult to determine how to psychiatrically treat patients with psychotic disorders if a clozapine trial failed [19]. In this patient, clozapine was abruptly discontinued from 175 mg twice per day without known side effects or withdrawal. However, further research into tapering clozapine or cross-tapering with another antipsychotic to prevent withdrawal symptoms and psychiatric decompensation would be helpful. Research into the most effective treatment options following the failure of clozapine would also be valuable for clinicians managing treatment-resistant patients who fail a trial of clozapine.

## Conclusions

Hepatotoxicity is a rare side effect of clozapine, with limited case reports in the literature. Further research in larger populations is needed to better understand the association of clozapine use with drug-induced liver injury. Additionally, research aimed at uncovering the mechanism of this injury use would be beneficial in identifying patients at risk for clozapine-induced hepatotoxicity and proper monitoring and management protocols for these patients. Due to its rarity, there is currently little guidance on how to detect, monitor, and manage these patients. Therefore, it is important for clinicians to recognize the symptoms of hepatotoxicity and order appropriate workup if liver injury is suspected. In severe symptomatic cases or fulminant liver failure, clozapine should be discontinued immediately. However, further research is needed to determine the most appropriate way to discontinue clozapine in patients with less severe cases of hepatotoxicity who may be prone to psychiatric decompensation or withdrawal effects if clozapine is discontinued abruptly.
